# Positive effects of molybdenum on the biomineralization process on the surface of low-alloy steel catalyzed by *Bacillus subtilis*

**DOI:** 10.3389/fmicb.2024.1428286

**Published:** 2024-08-30

**Authors:** Zhangwei Guo, Qun Feng, Na Guo, Yansheng Yin, Tao Liu

**Affiliations:** ^1^College of Ocean Science and Engineering, Shanghai Maritime University, Shanghai, China; ^2^Engineering Technology Research Center for Corrosion Control and Protection of Materials in Extreme Marine Environment, Guangzhou Maritime University, Guangzhou, China

**Keywords:** bioprecipitation, molybdenum, corrosion, molecular mechanisms, RNAseq

## Abstract

The adhesion of microorganisms and the subsequent formation of mineralized layers in biofilms are of great significance in inhibiting the corrosion of metal materials. In this work, we found that the adhesion and subsequent mineralization of *Bacillus subtilis* on the surface of low-alloy steel are influenced by the molybdenum in the material. The addition of molybdenum will lead to increased adhesion of *B. subtilis* on the material surface, and the subsequent biomineralization ability has also been improved. Through transcriptome and physiological and biochemical tests, we found that molybdenum can affect the chemotaxis, mobility and carbonic anhydrase secretion related genes of *B. subtilis*, and then affect the formation and mineralization of the biofilm of *B. subtilis*.

## 1 Introduction

*Bacillus subtilis*, a gram-positive bacterium species of *Bacillus*, has a single cell size of 0.7–0.8 × 2–3 μm, no capsule, and peritrichous flagella and is motile. *B. subtilis* can form endogenous spores under stress and resist various adverse environments; therefore, it has a wide survival range and has been observed in soil, freshwater, and seawater (Mohsin et al., 2021). As a gram-positive bacterium, *B. subtilis* has the functionality of biological mineralization (Yan et al., 2021; Wightman and Fein, 2005; Mudgil et al., 2018; Lin et al., 2015; Guo et al., 2019; Chalia et al., 2017), with its abundant extracellular products providing many nucleation sites for mineralization (Yin et al., 2020; Fein et al., 2002; Keren-Paz et al., 2022). More importantly, *B. subtilis* can secrete carbonic anhydrase (CA) that is a ubiquitous enzyme. It can efficiently catalyze the reversible reactions (Han et al., 2019; Mukherjee and Venkata Mohan, 2021; Barabesi et al., 2007) of CO_2_ and H_2_O to generate HCO_3_^−^and H^+^, and plays a crucial role in the process of biomineralization. As a result, this property of *B. subtilis* has been widely used in the preparation of biomineralized CaCO_3_ crystals (Song et al., 2019; Perito et al., 2018; Marvasi et al., 2010), as adsorptive materials (Arias et al., 2018), catalytic materials, ceramic materials, protective layers for sensitive materials (such as enzymes, proteins), and drug release materials, with broad application prospects in the fields of chemical, environmental protection, biological, medical, and building material industries (Kang et al., 2021). It can also be used in engineering, such as concrete micro-crack repair (Mondal and Ghosh, 2021; Huynh et al., 2019; Feng et al., 2021; Huynh et al., 2022; Mahmood et al., 2022), building restoration and protection (Perito et al., 2014), and preparation of calcium carbonate micro- and nano-particles (Sazanova et al., 2020). Thus, its role in the protection and restoration of stone surfaces (Perito et al., 2014; Sazanova et al., 2020; Shim et al., 2011), foundation stabilization, earthquake prevention, and the capture of radionuclides and heavy metal ions (Johnson and Fein, 2019) should not be disregarded (Arias et al., 2020).

The calcium carbonate layer formed by microbial mineralization on metal surfaces can protect materials and prevent metal material corrosion. As a green, environmentally friendly, and self-healing anti-corrosion method, this approach has attracted the attention of scholars. A previous study has reported that the presence and concentration of Mo in metal materials affects the adhesion of microorganisms, thus affecting the subsequent mineralization or corrosion mediated by the microorganisms (Guo et al., 2022; Guo et al., 2019). However, although the *B. subtilis* bacterium has an excellent mineralization ability, the effect of metal alloy elements on the adhesion and mineralization performance of *B. subtilis* has not been reported. Moreover, in the CA-induced mineralization process, the CA activity will be affected by a number of factors, such as temperature, pH, and Ca^2+^ concentration. Also, whether metals affect the expression of CA remains unclear. Therefore, a study on the effects of elements present in metal materials on *B. subtilis* and its CA activity is of great significance to further clarify the mechanism of *B. subtilis* mineralization, providing an important scientific basis for the preparation of biomineralization coatings on metal surfaces. In this study, low-alloy steels containing Mo with different gradient concentrations were designed to study the influence of changes in Mo content on the adhesion and mineralization behavior of *B. subtilis*. Finally, the influence mechanism of Mo on the adhesion and mineralization behavior of *B. subtilis* was investigated through transcriptome analysis.

## 2 Materials and methods

### 2.1 Steel sample

The steel sample used in the test was low-alloy steel, and its composition is shown in Supplementary Table S1. The steel samples were processed into 10 × 10 mm square samples by wire cutting, and then we used 50–1,200 grit sandpaper to polish the samples in sequence. The steel samples were soaked in anhydrous ethanol and acetone, and then washed under ultrasonication for 15 min and dried with nitrogen. The samples were placed on a clean workbench for 1 h of ultraviolet sterilization before use.

### 2.2 Strain and culture

The *B. subtilis* used in this study was supplied by Marine Culture Collection of China (No. 1A14806), it could mineralize on the surfaces of metal materials, thereby inhibiting metal material corrosion. The strain was stored in a −80°C freezer. The medium was 2216E medium (Qingdao Hopebio), and 37.5 g of the medium was dissolved in deionized water to prepare the experimental medium. 2216E medium: 19.45 g/L NaCl, 5.98 g/L MgCl_2_, 5.0 g/L Peptone, 3.24 g/LNa_2_SO_4_, 1.8 g/L CaCl_2_, 1.0 g/L yeast extract powder, 0.55 g/L KCl, 0.16 g/L Na_2_CO_3_, 0.1 g/L FeC_6_H_5_O_7_, 0.08 g/L KBr, 0.034 g/L SrCl_2_, 0.022 g/L H_3_BO_3_, 0.008 g/L NaH_2_PO_4_, 0.004 g/L Na_2_SiO_3_, 0.0024 g/L NaF, 0.0016 g/L NH_4_NO_3_. After the culture medium was sterilized at 121°C for 20 min, we cultured 100 μL of the bacterial solution overnight and inoculated the culture medium (200 mL). Then, the steel sample was placed in the culture medium and cultured in a light incubator at a temperature of 30°C and a shaker speed of 120 r/min. The growth of the bacterium was monitored by plate counting, using a series of dilutions (in a total volume of 100 μL). The concentration of viable cells was calculated based on the number of colonies grown in each well on the plate. In addition, pH and dissolved oxygen levels were measured once a day in the cultures. A pH meter (Sartorius, Germany) and a dissolved oxygen meter (Inesa JPBJ-611Y, China) were used to obtain the respective measurements. Throughout the experimental period, the changes in calcium ion concentration were measured in filtered samples (0.22 μm) using an inductively coupled plasma mass spectrometer (PerkinElmer NexION 2,200, United States).

### 2.3 Corrosion morphology characterization

After 14 days of cultivation, the steel samples were removed, washed with PBS, and soaked in 2.5% glutaraldehyde solution for 2 h. Afterward, the steel samples were dried with alcohol (stepwise in: 30, 50, 70, 80, 90, and 100% v/v, 15 min each) and then dried under pure nitrogen. The corrosion morphology of the steel samples was observed by scanning electron microscope (SEM, ZEISS Gemini 300, Germany), and X-ray diffractometer (XRD, Nalytical X’Pert PRO, Netherlands) was used to identify the corrosion product components on the sample surfaces (Cu-Ka radiation source at 40 kV and 10 mA with 2θ = 20–90°). The adhesion of the bacterial biofilm on the steel surface was investigated using acridine orange as staining agent, with the bacterial cells showing green fluorescence under an inverted fluorescence microscope (after 15 min staining, at 502 nm maximum excitation wavelength). Then, the specimens were washed successively with concentrated hydrochloric acid, saturated sodium bicarbonate, and deionized water to clean the product layer on the steel surface. After the samples were air-dried, pitting on the material surface was observed by an optical profiler (Bruker ContourGT, Germany).

### 2.4 Electrochemistry test

An electrochemistry test was conducted using a three-electrode system, where the sample served as the working electrode, the saturated calomel electrode was the reference electrode, the platinum electrode was the opposite electrode, and the test solution was 2216E medium. Before impedance testing was conducted, the sample was soaked for 60 min, and then the OCP test was performed. After a stable open-circuit potential was obtained, the EIS test was performed by an electrochemical workstation (Gamry Interface 100E, United States), using a test frequency range of 10^5^–10^−2^ Hz, and the interference amplitude was ±10 mV. ZSimpWin analysis software was used.

### 2.5 RNA-seq test

A biofilm developed on the low-alloy steel surface, and biofilm samples were collected after 2 days of inoculation. After removal from the media, the coupons were quickly and gently washed in 0.85% NaCl buffer at 0°C to remove the contaminated suspended cells and ultrasonically treated in 0.85% NaCl buffer at 0°C for 2 min to collect the biofilm cells from the metal surface. The buffer containing biofilm cells was centrifuged at 10000 rpm and −2°C for 3 min, then the supernatant was discarded and the precipitated biofilm cells were resuspended in 6 mL of 0.85% NaCl buffer at 0°C, transferred to a cold bead stirrer tube, and centrifuged at 10000 rpm and room temperature for 15 s. Then, the cell particles were immediately frozen in a dry ice ethanol bath, and subsequently the samples were sent to Shanghai Majorbio Bio-pharm Technology Co., Ltd., for transcriptome testing.

## 3 Results

### 3.1 Bacterial adhesion to the surface of low-alloy steel

**Figure 1:**
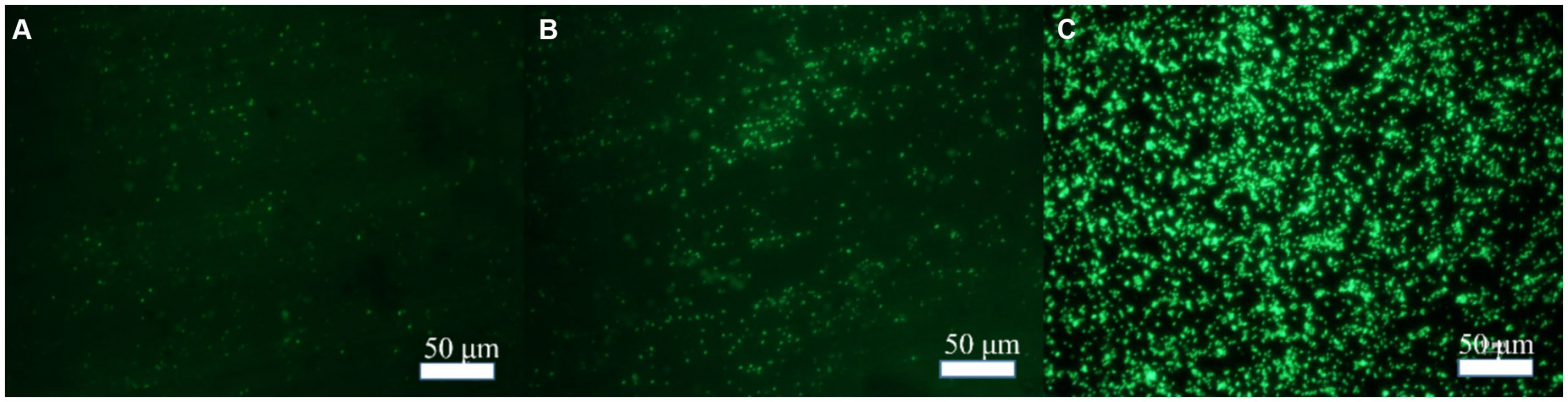
Bacterial adhesion on blank steel and steel samples containing Mo. **(A)** Blank steel, **(B)** 0.4 wt% Mo steel, and **(C)** 1.0 wt% Mo steel.

In order to explore the effect of Mo on the adhesion of *B. subtilis* on the low-alloy steel surface and the formation of microbial film, the surfaces of the low-alloy steel samples with different Mo element contents were characterized by an inverted fluorescence microscope, after 3 days of immersion (Figure 1). The number of bacteria on the low-alloy steel surface without Mo (Figure 1A) elements was significantly lower than that of low-alloy steel containing 0.4 wt% Mo elements (Figure 1B). The number of bacteria attached to the low-alloy steel containing 1.0 wt% Mo elements (Figure 1C) was decisively the highest. Therefore, it can be seen that the adhesion number of bacteria on the low-allow steel surface increases with the increase of Mo content. According to our previous study (Guo et al., 2022; Guo et al., 2019), this was possibly due to the presence of Mo elements, which affected the chemotaxis of the bacteria and thus increased their quantity on the steel surface and affected their mineralization.

### 3.2 Surface morphology of low-alloy steel

**Figure 2:**
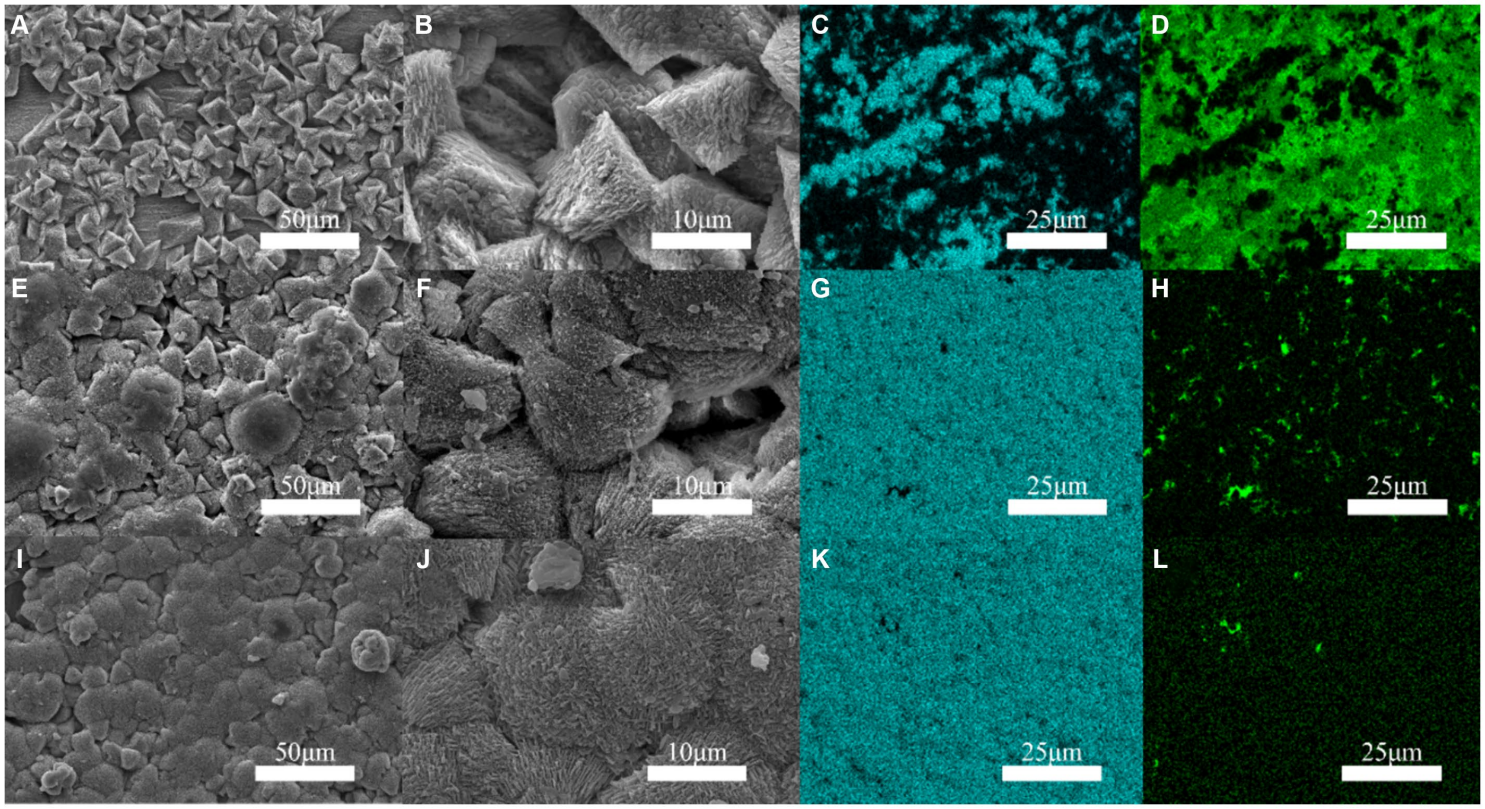
Surface mineralization of the blank steel and Mo-containing steel. **(A–D)** Blank steel, **(E–H)** 0.4 wt% Mo steel, **(I–L)** 1.0 wt% Mo steel.

Figure 2 indicates that the occurrence of triangular calcite structures raised with increasing Mo content, based on a comparison of the surface morphology of materials containing increasing Mo (Figures 2A,E,I), with the greatest number of mineralized products being observed on the low-alloy steel surface with 1.0 wt% Mo elements (Figures 2I,J). According to the mapping diagram, with blue representing the content of Ca elements on the surface, we observed that the content of Ca elements on the low-alloy steel surface rose with increasing Mo content in the low-alloy steel, which further supported the observation of elevated mineralized products on the steel surface. Green represented the content of Fe elements on the surface, and the results indicate that Fe content decreased with increasing Mo content, showing that both the amount of corrosion products and the area of uncovered steel surface decreased, while the amounts of mineralized products gradually increased.

**Figure 3:**
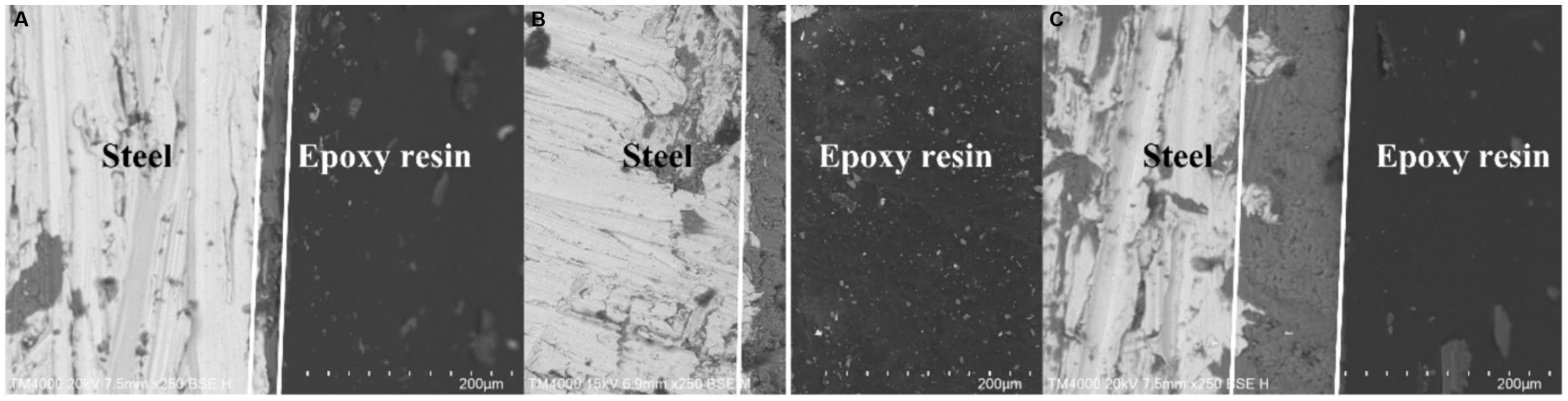
Surface mineralized thicknesses of the blank steel and steel containing Mo. **(A)** Blank steel (19 μm), **(B)** 0.4 wt% Mo steel (42 μm), **(C)** 1.0 wt% Mo steel (106 μm).

It is apparent in Figure 3 that the thickness of the mineralized product layer on the low-alloy steel surface without Mo elements (Figure 3A) was thinner than that of low-alloy steel with Mo elements (Figures 3B,C), which also proved that the thickness of the mineralized product layer on the low-alloy steel surface increases with the increase of Mo element content.

**Figure 4:**
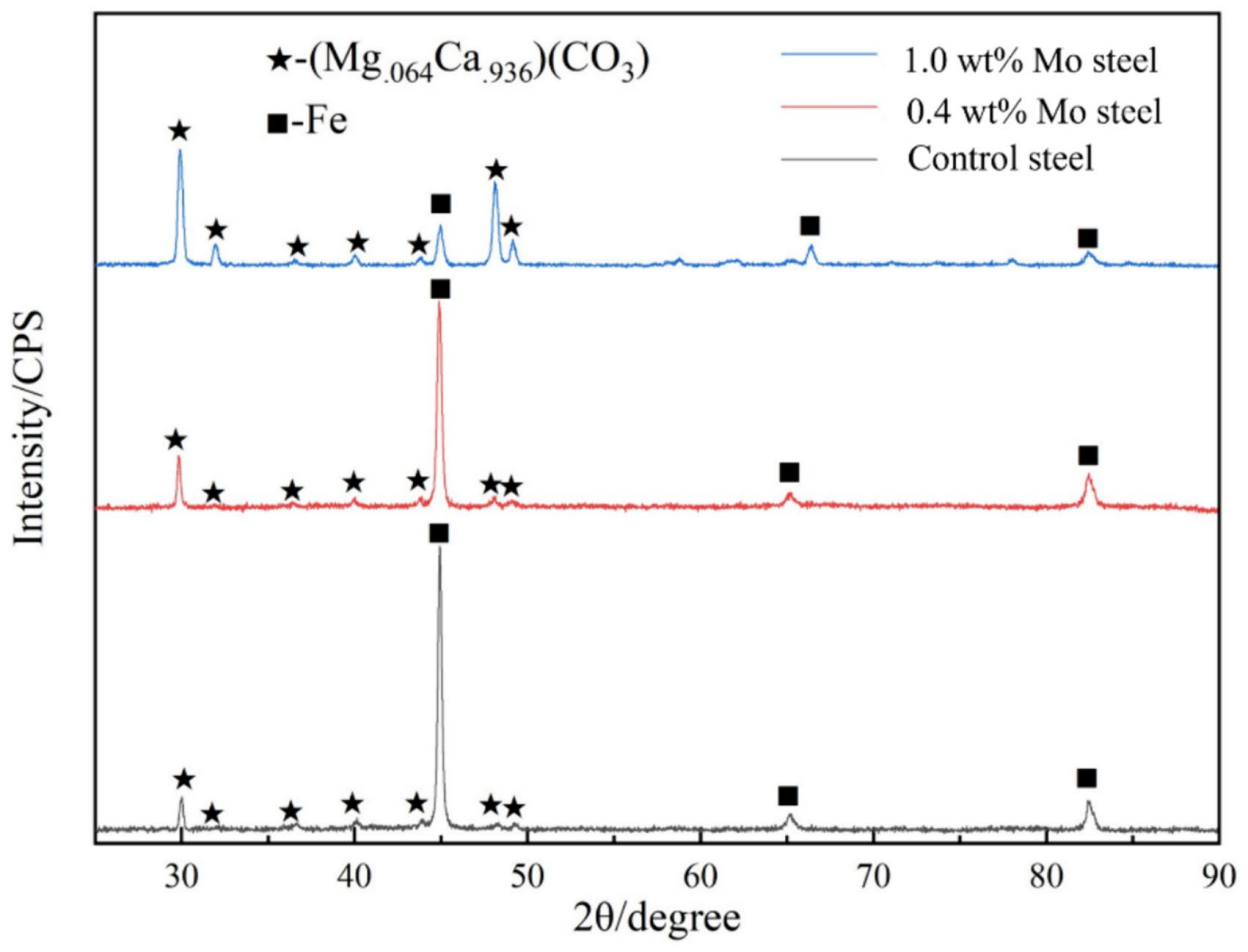
XRD patterns of the surface mineralized components of the blank steel and Mo steel.

Figure 4 shows the X-ray diffraction patterns results. As shown in the figure, compared with the low-alloy steel surface without Mo, the surface of the low-alloy steel with a higher Mo content was greater (Mg._064_Ca._936_) (CO_3_), with a thicker mineralized product layer, as shown also in Figure 3.

### 3.3 Surface pitting

**Figure 5:**
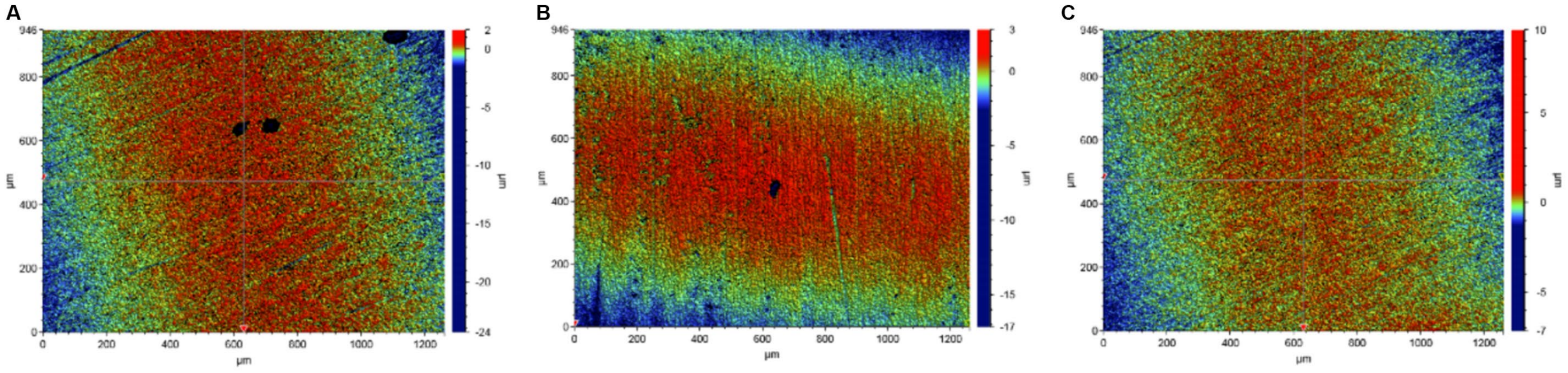
Corrosion of the blank steel and steel containing Mo in the presence of *B. subtilis*. **(A)** Blank steel, **(B)** 0.4 wt% Mo steel, **(C)** 1.0 wt% Mo steel.

As shown in the Figure 5, more extensive pitting was observed on the low-alloy steel surface without Mo elements (Figure 5A), compared with the low-alloy steel containing Mo, and with an increase in Mo content, pitting essentially disappeared (1.0 wt% Mo). Supplementary Figure S1 shows the scanning electron microscope images and pitting corrosion after 14 days of immersion in a sterile solution. We observed that the addition of Mo had no effect on corrosion under sterile conditions. In previous work (Guo et al., 2022; Guo et al., 2019), we compared pitting, weight loss, and electrochemical data in sterile environments, the addition of molybdenum did not alter the corrosion resistance of low-alloy steel.

### 3.4 *Bacillus subtilis* growth kinetics

**Figure 6:**
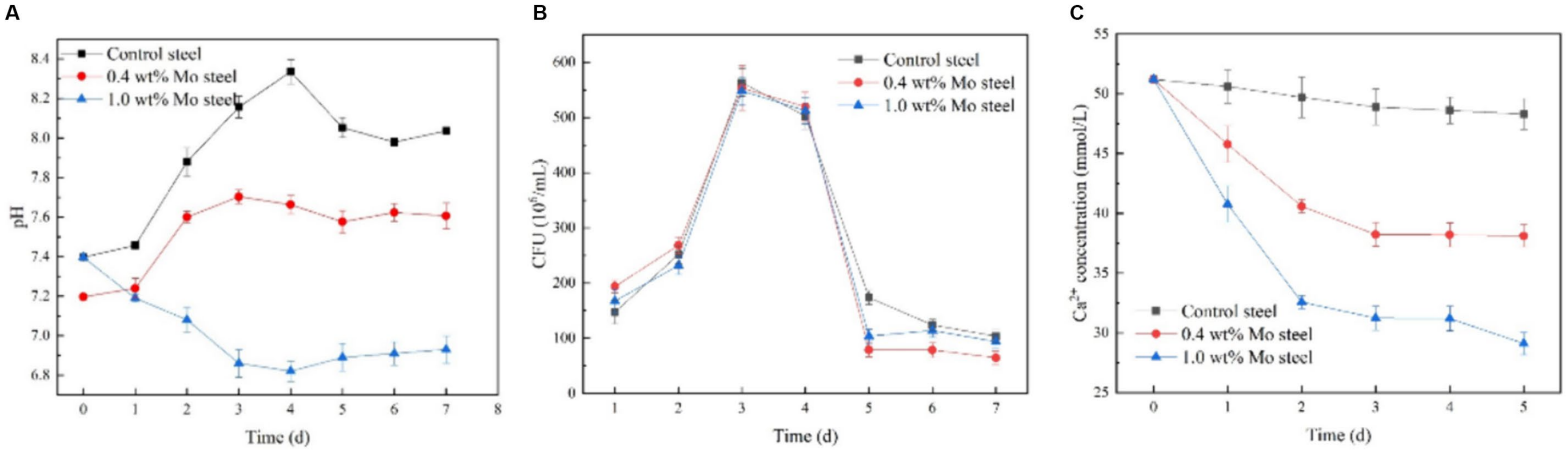
Growth and physiological and biochemical analysis of *B. subtilis*. **(A)** pH, **(B)** growth curve, **(C)** Ca^2+^ content in the solution.

Figure 6 shows kinetics of the bacterial growth. As indicated by the solution pH change (Figure 6A), pH value decreased with increasing Mo content in the low-alloy steel, which was ascribed to a series of biochemical reactions. The total number of *B. subtilis* colonies (Figure 6B) was not significantly affected by the Mo content. However, the change in Ca^2+^ concentration in the solution (Figure 6C) decreased with increasing Mo content, which supported the hypothesis that the increase in Mo content increased the formation of mineralized products on the sample surface, as this process consumes Ca^2+^ in the solution.

### 3.5 Electrochemical impedance

**Figure 7:**
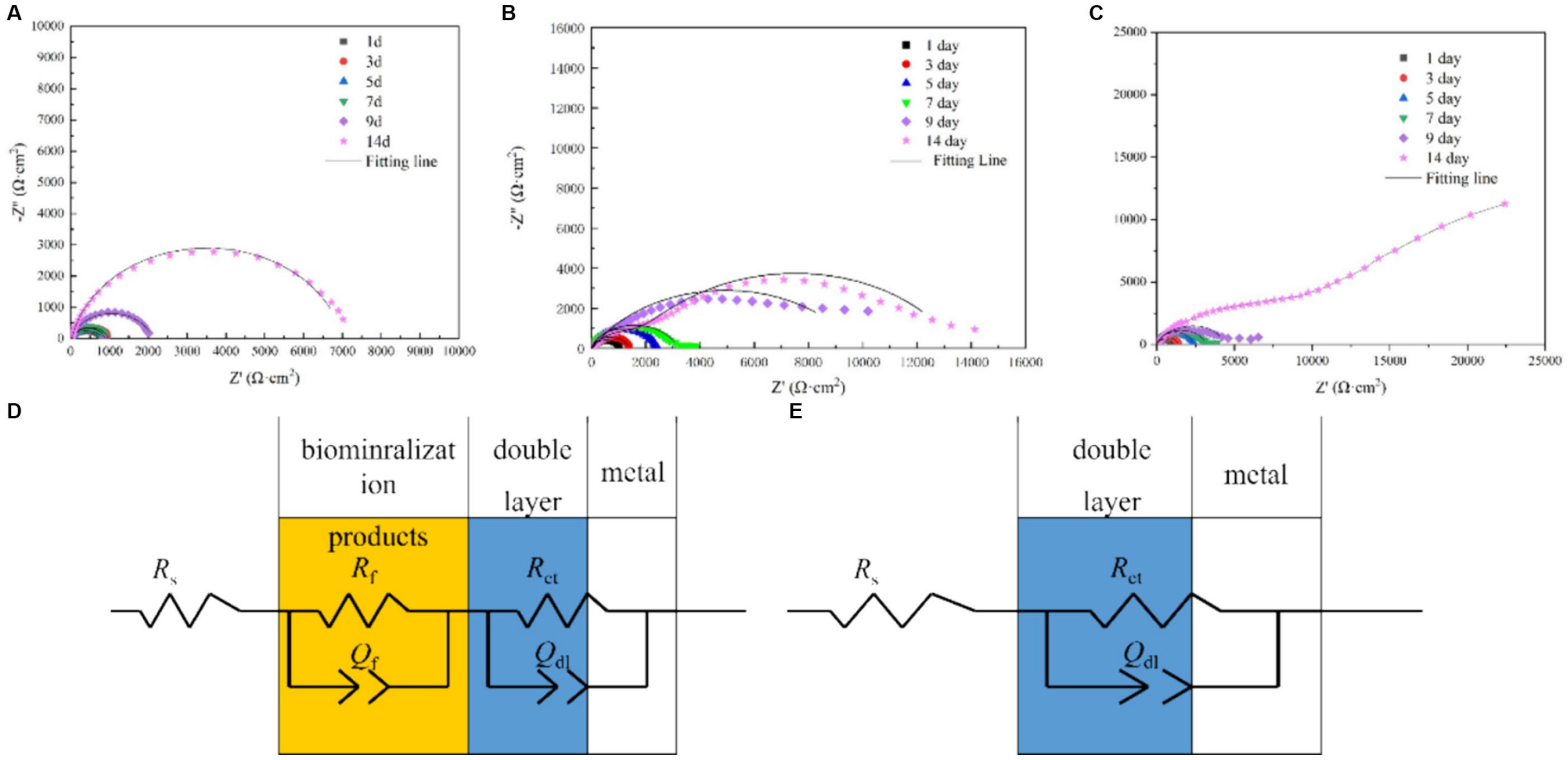
EIS spectra of the blank steel and steel containing Mo in the presence of *B. subtilis*. **(A)** Blank steel, **(B)** 0.4 wt% Mo steel, **(C)** 1.0 wt% Mo steel, and **(D,E)** corresponding fitting circuits.

Figure 7 shows the electrochemical impedance test results. As shown in the figure, the electrochemical impedance curves formed incomplete semicircles, and the radius changed with different Mo element contents. The radius of the impedance curve of the low-alloy steel without Mo elements (Figure 7A) during the immersion process was significantly smaller than that of the low-alloy steel with 0.4 wt% Mo element (Figure 7B) and low-allow steel containing 1.0 wt% Mo element (Figure 7C). Similarly, the radius of the impedance curve rose with increasing Mo content, correlating with the electrochemical impedance value. Electrochemical impedance parameters fitted from the measured impedance plots in Figure 7 are listed in Supplementary Table S2. Thus, with rising Mo content, the impedance increases, as well as the thickness of the surface mineralized product layer, which corresponded to the results obtained from the above characteristic maps. In general, the thicker and denser mineralized layers are the more electron transfer on the material surface is hindered and conductivity lowered (Guo et al., 2019).

### 3.6 Transcriptome analysis results

**Figure 8:**
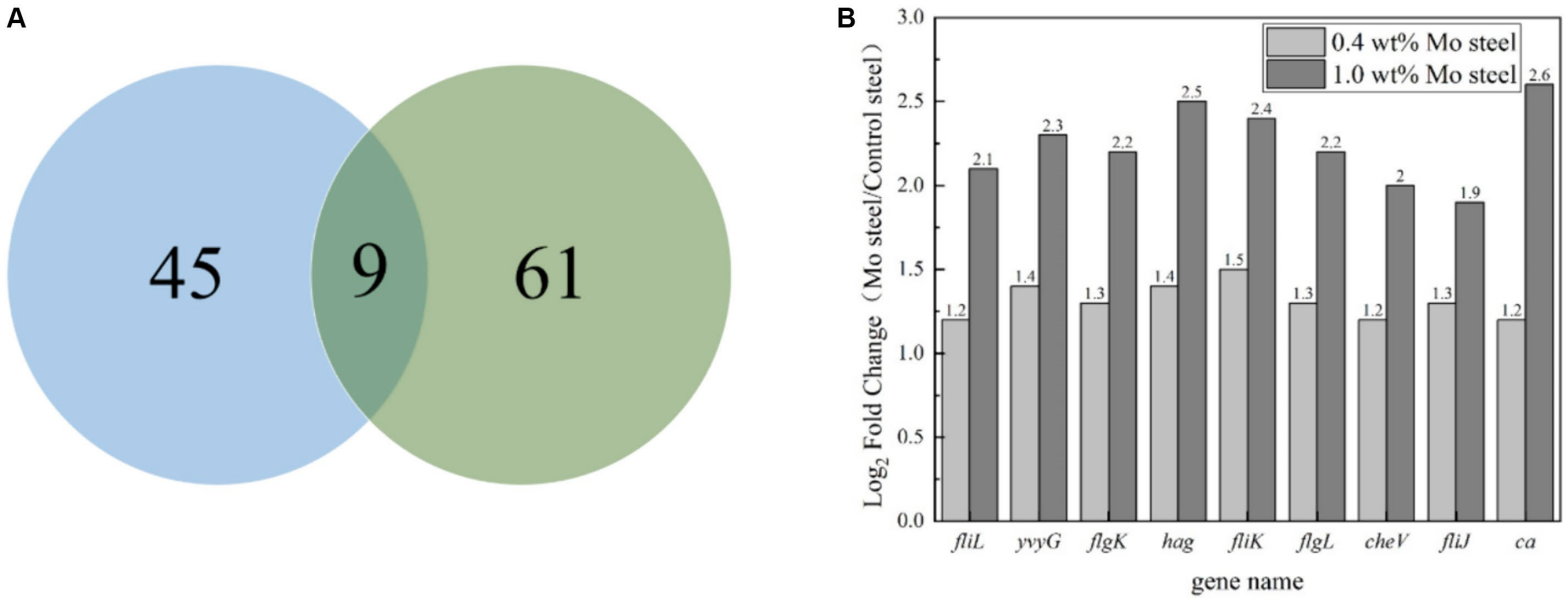
Gene regulation analysis of blank steel and steel containing Mo on *B. subtilis*. **(A)** Venn diagram, **(B)** differential gene analysis diagram.

Figure 8 summarizes the observed differences in gene expression. Based on findings of previous studies (Guo et al., 2022), we mainly focused on bacterial chemotaxis and flagellar pathways. Figure 8A shows a Venn diagram, with nine common differential genes in the experimental group. As shown in Figure 8B, *fliL*, *yvy*G, *flg*K, *hag*, *fli*K, *flg*L, *fli*J and *ca* genes were upregulated in cells collected from the steel surface with elevated Mo content.

## 4 Discussion

The presented study investigated regulatory mechanisms and factors influencing biomineralization on metal material surfaces mediated by *B. subtilis*, showing an important direction for research supporting future applications of biomineralization in corrosion prevention. In our previous study, 1.0 wt% Mo content regulated the attachment of *Pseudoalteromonas lipolytica* (Guo et al., 2019). In this work, *fliL*, *yvy*G, *flg*K, *hag*, *fli*K, *flg*L, *fli*J and *ca* genes were upregulated by Mo in low-alloy steel, with *fli*L, *yvy*G, *flg*K, hag, *fli*K, *flg*L, and *fli*J being genes involved in the assembly of bacterial flagella, and *che*V affecting the chemotaxis of bacteria. In general, adhesion of bacteria is greatly affected by the assembly of flagella and chemotaxis. Therefore, this study indicated that Mo affected flagella assembly and bacterial chemotaxis. Chemotaxis refers to the process of cells responding to a chemical gradient by the movement towards a more favorable environment (Chen et al., 2024), with methyl-accepting chemotaxis protein (Mcp) being one of key signalling molecules (Song et al., 2024). Mcps interact with other chemotactic molecules, which leads to the enhancement of phosphorylation of CheV and the charge of the Mot protein subunit, thus controlling the direction of the movement by increasing the rotation rate of the flagella. In this study, we reported that bacteria adhered more to the steel surface containing Mo, compared to Mo-free steel, by the process of flagella-assisted chemotaxis, which is consistent with our previous reports (Guo et al., 2022; Guo et al., 2019). It is noteworthy that in the transcriptome test performed in this study, the *ca* gene was also upregulated in the presence of Mo. Carbonic anhydrase is a common enzyme in bacteria, which can catalyze a series of physiological and biochemical reactions (Baidya et al., 2024). Its role involves combining carbon dioxide and water to form bicarbonate and hydrogen ions (Zheng et al., 2023):


$${CO}_{2}\;\left(aq\right)+H_{2}O\to {{HCO}_{3}}^{-}+H^{+}\text{,}$$

which will reduce the pH value of the solution, which is consistent with our pH test results. At the same time, bicarbonate can form carbonate, and then combine with the Ca ions in the solution (Zheng et al., 2023):


$${{HCO}_{3}}^{-}\to H^{+}+{{CO}_{3}}^{2-}\text{,}$$

$${Ca}^{2+}+{{CO}_{3}}^{2-}\to {\mathrm{CaCO}}_{3}↓\text{,}$$

which will form a denser mineralized layer. This was evidenced by the continuous decreases in pH and Ca content in solution. The main conclusion based on the transcriptomic analysis performed is that both chemotaxis and CA strengthened the mineralization ability of *B. subtilis* on the surface of steel with Mo content.

## 5 Conclusion

In this study, we report that Mo in low-alloy steel had a regulatory effect on the gram-positive bacterium *B. subtilis*. The expression of the chemotactic gene and *ca* gene in *B. subtilis* was enhanced in the presence of Mo, which improved the adhesion ability of the bacterium and enhanced the mineralization of calcium carbonate. Furthermore, mineralized products have shown to be stable and environmentally friendly; thus, this process controlled by *ca* gene has become one of the most promising technologies for CO_2_ capture. Future research should focus on the diversity of biomineralization patterns of different microbial species under specific environmental conditions, and investigate how these patterns are influenced by gene regulation and external environment.

## Data Availability

The datasets presented in this study can be found in online repositories. The names of the repository/repositories and accession number(s) can be found in the article/Supplementary material.

## References

[ref1] Arias D. Rivas M. Guiñez R. Cisternas L. A. (2018). Modeling the calcium and magnesium removal from seawater by immobilized biomass of ureolytic bacteria *Bacillus subtilis* through response surface methodology and artificial neural networks, desalination. Water Treat 118, 294–303. doi: 10.5004/dwt.2018.22665

[ref2] Arias D. Villca G. Pánico A. Cisternas L. A. Jeldres R. I. González-Benito G.. (2020). Partial desalination of seawater for mining processes through a fluidized bed bioreactor filled with immobilized cells of *Bacillus subtilis* LN8B. Desalination 482:114388. doi: 10.1016/j.desal.2020.114388

[ref3] Baidya P. Zhang M. Xiao Y. Zhang H. Yu L. Li W. (2024). Genetically engineered whole-cell biocatalyst for efficient CO_2_ capture by cell surface display of carbonic anhydrase from *Bacillus cereus* GLRT202 on *Escherichia coli*. Biochem. Eng. J. 211:109446. doi: 10.1016/j.bej.2024.109446

[ref4] Barabesi C. Galizzi A. Mastromei G. Rossi M. Tamburini E. Perito B. (2007). *Bacillus subtilis* gene cluster involved in calcium carbonate biomineralization. J. Bacteriol. 189, 228–235. doi: 10.1128/JB.01450-06, PMID: 17085570 PMC1797216

[ref5] Chalia S. Baskar S. Minakshi P. Baskar R. Ranjan K. (2017). Biomineralization abilities of *Cupriavidus* strain and *Bacillus subtilis* strains *in vitro* isolated from speleothems, Rani cave, Chhattisgarh, India. Geomicrobiol J. 34, 737–752. doi: 10.1080/01490451.2016.1257663

[ref6] Chen M. Trotter V. V. Walian P. J. Chen Y. Lopez R. Lui L. M.. (2024). Molecular mechanisms and environmental adaptations of flagellar loss and biofilm growth of *Rhodanobacter* under environmental stress. ISME J. 93:e151. doi: 10.1093/ismejo/wrae151, PMID: 39113613 PMC11410051

[ref7] Fein J.B. Scott S. Rivera N., The effect of Fe on Si adsorption by Bacillus subtilis cell walls: Insights into non-metabolic bacterial precipitation of silicate minerals, (2002), Available at: www.elsevier.comrlocaterchemgeo, 182, 265–273

[ref8] Feng J. Chen B. Sun W. Wang Y. (2021). Microbial induced calcium carbonate precipitation study using *Bacillus subtilis* with application to self-healing concrete preparation and characterization. Constr. Build. Mater. 280:122460. doi: 10.1016/j.conbuildmat.2021.122460

[ref9] Guo Z. Chai Z. Liu T. Gao S. Hui X. Zhang C.. (2022). *Pseudomonas aeruginosa*-accelerated corrosion of Mo-bearing low-alloy steel through molybdenum-mediating chemotaxis and motility. Bioelectrochemistry 144:108047. doi: 10.1016/j.bioelechem.2021.108047 35007894

[ref10] Guo Z. Pan S. Liu T. Zhao Q. Wang Y. Guo N.. (2019). *Bacillus subtilis* inhibits *Vibrio natriegens*-induced corrosion via biomineralization in seawater. Front. Microbiol. 10:e1111. doi: 10.3389/fmicb.2019.01111 PMC6536734 31164881

[ref11] Guo Z. Wang W. Guo N. Zeng Z. Liu T. Wang X. (2019). Molybdenum-mediated chemotaxis of *Pseudoalteromonas lipolytica* enhances biofilm-induced mineralization on low alloy steel surface. Corros. Sci. 159:108123. doi: 10.1016/j.corsci.2019.108123

[ref12] Han Z. Wang J. Zhao H. Tucker M. E. Zhao Y. Wu G.. (2019). Mechanism of biomineralization induced by *Bacillus subtilis* J2 and characteristics of the biominerals. Fortschr. Mineral. 9:40218. doi: 10.3390/min9040218

[ref13] Huynh N. N. T. Imamoto K. I. Kiyohara C. (2019). A study on biomineralization using *Bacillus subtilis* natto for repeatability of self-healing concrete and strength improvement. J. Adv. Concr. Technol. 17, 700–714. doi: 10.3151/jact.17.700

[ref14] Huynh N. N. T. Imamoto K. I. Kiyohara C. (2022). Biomineralization analysis and hydration acceleration effect in self-healing concrete using *Bacillus subtilis* natto. J. Adv. Concr. Technol. 20, 609–623. doi: 10.3151/jact.20.609

[ref15] Johnson C. R. Fein J. B. (2019). A mechanistic study of au(III) removal from solution by *Bacillus subtilis*. Geomicrobiol J. 36, 506–514. doi: 10.1080/01490451.2019.1573279

[ref16] Kang S. Y. Pokhrel A. Bratsch S. Benson J. J. Seo S. O. Quin M. B.. (2021). Engineering *Bacillus subtilis* for the formation of a durable living biocomposite material. Nat. Commun. 12:7133. doi: 10.1038/s41467-021-27467-2, PMID: 34880257 PMC8654922

[ref17] Keren-Paz A. Maan H. Karunker I. Olender T. Kapishnikov S. Dersch S.. (2022). The roles of intracellular and extracellular calcium in *Bacillus subtilis* biofilms. IScience 25:104308. doi: 10.1016/j.isci.2022.104308, PMID: 35663026 PMC9160756

[ref18] Lin W. Huang Z. Li X. Liu M. Cheng Y. (2015). Bio-remediation of acephate–Pb(II) compound contaminants by *Bacillus subtilis* FZUL-33. J. Environ. Sci. 45, 94–99. doi: 10.1016/j.jes.2015.12.010, PMID: 27372122

[ref19] Mahmood F. Rehman S. K. Jameel M. Riaz N. Javed M. F. Salmi A.. (2022). Self-healing bio-concrete using *Bacillus subtilis* encapsulated in Iron oxide nanoparticles. Materials 15:7731. doi: 10.3390/ma15217731, PMID: 36363323 PMC9656118

[ref20] Marvasi M. Visscher P. T. Perito B. Mastromei G. Casillas-Martínez L. (2010). Physiological requirements for carbonate precipitation during biofilm development of *Bacillus subtilis* etfA mutant. FEMS Microbiol. Ecol. 71, 341–350. doi: 10.1111/j.1574-6941.2009.00805.x, PMID: 20059546

[ref21] Mohsin M. Z. Omer R. Huang J. Mohsin A. Guo M. Qian J.. (2021). Advances in engineered *Bacillus subtilis* biofilms and spores, and their applications in bioremediation, biocatalysis, and biomaterials. Synth. Syst. Biotechnol. 6, 180–191. doi: 10.1016/j.synbio.2021.07.002, PMID: 34401544 PMC8332661

[ref22] Mondal S. Ghosh A. (2021). Spore-forming *Bacillus subtilis* Vis-à-Vis non-spore-forming *Deinococcus radiodurans*, a novel bacterium for self-healing of concrete structures: a comparative study. Constr. Build. Mater. 266:121122. doi: 10.1016/j.conbuildmat.2020.121122

[ref23] Mudgil D. Baskar S. Baskar R. Paul D. Shouche Y. S. (2018). Biomineralization potential of *Bacillus subtilis*, *Rummeliibacillus Stabekisii* and *Staphylococcus Epidermidis* strains in vitro isolated from speleothems, Khasi Hill caves, Meghalaya, India. Geomicrobiol J. 35, 675–694. doi: 10.1080/01490451.2018.1450461

[ref24] Mukherjee T. Venkata Mohan S. (2021). Metabolic flux of *Bacillus subtilis* under poised potential in electrofermentation system: gene expression vs product formation. Bioresour. Technol. 342:125854. doi: 10.1016/j.biortech.2021.125854, PMID: 34537531

[ref25] Perito B. Casillas L. Marvasi M. (2018). Factors affecting formation of large calcite crystals (≥1 mm) in *Bacillus subtilis* 168 biofilm. Geomicrobiol J. 35, 385–391. doi: 10.1080/01490451.2017.1377788

[ref26] Perito B. Marvasi M. Barabesi C. Mastromei G. Bracci S. Vendrell M.. (2014). A *Bacillus subtilis* cell fraction (BCF) inducing calcium carbonate precipitation: biotechnological perspectives for monumental stone reinforcement. J. Cult. Herit. 15, 345–351. doi: 10.1016/j.culher.2013.10.001

[ref27] Sazanova K. V. Frank-Kamenetskaya O. V. Vlasov D. Y. Zelenskaya M. S. Vlasov A. D. Rusakov A. V.. (2020). Carbonate and oxalate crystallization by interaction of calcite marble with *Bacillus subtilis* and *Bacillus subtilis*–aspergillus Niger association. Crystals (Basel) 10, 1–16. doi: 10.3390/cryst10090756

[ref28] Shim H. W. Jin Y. H. Seo S. D. Lee S. H. Kim D. W. (2011). Highly reversible lithium storage in *Bacillus subtilis*-directed porous Co_3_O_4_ nanostructures. ACS Nano 5, 443–449. doi: 10.1021/nn1021605, PMID: 21155558

[ref29] Song J. Han B. Song H. Yang J. Zhang L. Ning P.. (2019). Nonreductive biomineralization of uranium by *Bacillus subtilis* ATCC–6633 under aerobic conditions. J. Environ. Radioact. 208–209:106027. doi: 10.1016/j.jenvrad.2019.106027, PMID: 31442938

[ref30] Song Q. Li X. Hou N. Pei C. Li D. (2024). Chemotaxis-mediated degradation of PAHs and heterocyclic PAHs under low-temperature stress by *Pseudomonas fluorescens* S01: insights into the mechanisms of biodegradation and cold adaptation. J. Hazard. Mater. 469:133905. doi: 10.1016/j.jhazmat.2024.133905, PMID: 38422734

[ref31] Wightman P. G. Fein J. B. (2005). Iron adsorption by *Bacillus subtilis* bacterial cell walls. Chem. Geol. 216, 177–189. doi: 10.1016/j.chemgeo.2004.11.008

[ref32] Yan H. Owusu D. C. Han Z. Zhao H. Ji B. Zhao Y.. (2021). Extracellular, surface, and intracellular biomineralization of *Bacillus subtilis* Daniel-1 Bacteria. Geomicrobiol J. 38, 698–708. doi: 10.1080/01490451.2021.1937406

[ref33] Yin X. Weitzel F. Griesshaber E. Fernández-Díaz L. Jimenez-Lopez C. Ziegler A.. (2020). Bacterial EPS in agarose hydrogels directs mineral Organization in Calcite Precipitates: species-specific biosignatures of *Bacillus subtilis*, *Mycobacterium phley*, *Mycobacterium smagmatis*, and *Pseudomonas putida* EPS. Cryst. Growth Des. 20, 4402–4417. doi: 10.1021/acs.cgd.0c00231

[ref34] Zheng T. Hou D. Leng W. Li P. Wei W. (2023). Preparation, characterization, and formation mechanism of different biological calcium carbonate (CaCO_3_) induced by *Bacillus mucilaginosus* and *Bacillus alcalophilus*. J. Nanopart. Res. 25:189. doi: 10.1007/s11051-023-05833-z

