# Supplementary material for: Positive effects of molybdenum on the biomineralization process on the surface of low-alloy steel catalyzed by Bacillus subtilis
Source: Front Microbiol. 2024 Aug 30;15:1428286. doi: 10.3389/fmicb.2024.1428286 (PMC11401046; doi:10.3389/fmicb.2024.1428286)
Supplement: Supplementary file 1 [file Data_Sheet_1.pdf]

Supplementary material

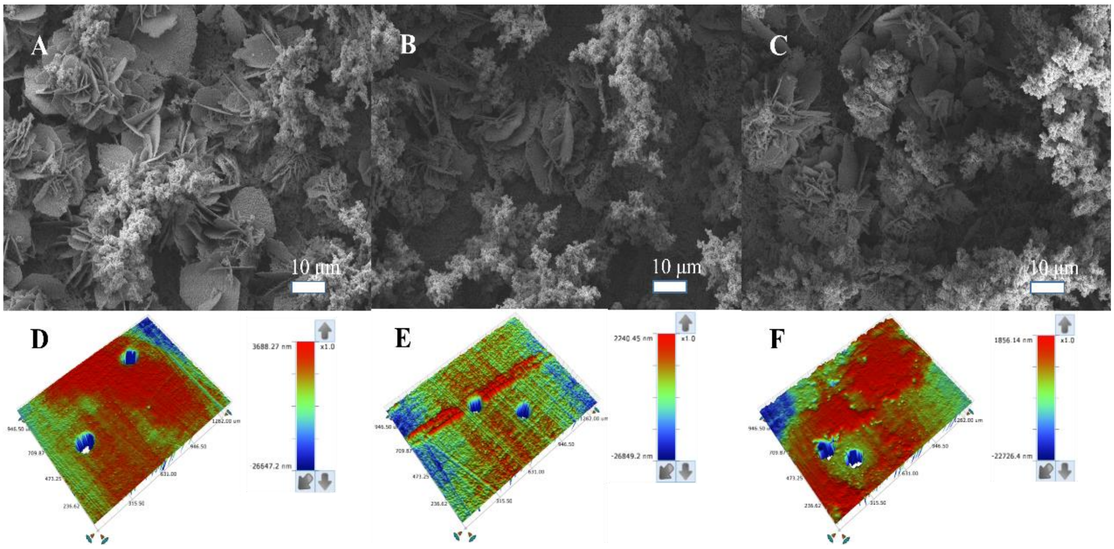

Figure S1. Corrosion of the blank steel and steel containing Mo under aseptic conditions. A and D: Blank steel, B and E: 0.4 wt% Mo steel, C and F: 1.0 wt% Mo steel.

Table S1. Composition of the steel

Element	Mn	Ni	Al	Cr	C	Mo	Fe
Control	1.52	0.70	0.04	0.15	0.05	0	Balance
0.4 wt% Mo	1.52	0.70	0.04	0.15	0.05	0.40	Balance
1.0 wt% Mo	1.52	0.70	0.04	0.15	0.05	1.00	Balance

Table S2. Electrochemical impedance parameters fitted from the measured impedance plots in Figure 7.

	Y_f ($S.sec^n/cm^2$)	n	R_f (Ω cm^2)	Y_{dl} ($S.sec^n/cm^2$)	n	R_{ct} (Ω cm^2)
Control-1d	1.011E-003	0.83	1.043E+003	1.114E-004	0.86	2.145E+003
Control-3d	1.180E-003	0.91	1.158E+003	1.169E-004	0.89	2.914E+003
Control-5d	1.137E-003	0.89	1.160E+003	1.357E-004	0.83	2.953E+000
Control-7d	1.354E-003	0.87	1.165E+003	1.267E-004	0.89	3.214E+003
Control-9d	2.025E-004	0.82	2.513E+003	3.563E-005	0.85	4.113E+003
Control-14d	2.012E-005	0.86	5.342E+003	3.652E-005	0.84	7.614E+003
0.4 wt%-1d	4.254E-004	0.75	7.021E+002	5.717E-004	0.89	8.112E+002
0.4 wt%-3d	5.613E-004	0.84	1.074E+003	1.006E-005	0.85	1.107E+003
0.4 wt%-5d	1.104E-005	0.71	2.151E+003	2.194E-005	0.89	2.112E+003
0.4 wt%-7d	2.813E-005	0.79	4.013E+003	4.308E-005	0.88	4.053E+003

0.4 wt%-9d	7.152E-005	0.88	8.173E+003	8.148E-005	0.71	1.101E+004
0.4 wt%-14d	1.012E-006	0.85	1.125E+004	1.173E-006	0.86	1.414E+004
1.0 wt%-1d	4.712E-004	0.85	1.012E+002	5.813E-004	0.88	9.122E+002
1.0 wt%-3d	5.864E-004	0.83	1.482E+003	1.216E-005	0.86	1.314E+003
1.0 wt%-5d	1.212E-005	0.87	2.349E+003	2.087E-005	0.88	2.241E+003
1.0 wt%-7d	2.471E-005	0.89	3.872E+003	4.115E-005	0.89	3.913E+003
1.0 wt%-9d	4.782E-005	0.83	5.561E+003	5.347E-005	0.82	7.583E+003
1.0 wt%-14d	1.712E-006	0.88	1.913E+004	2.822E-006	0.87	2.405E+004
